# The optimal algorithm for Multi-source RS image fusion

**DOI:** 10.1016/j.mex.2015.12.004

**Published:** 2015-12-21

**Authors:** Wei Fu, Shui-guang Huang, Zeng-shun Li, Hao Shen, Jun-shuai Li, Peng-yuan Wang

**Affiliations:** College of Electrical and Electronic Engineering of Tianhe College, Guangdong Polytechnical Normal University, Guangzhou 510540, China

**Keywords:** RS image fusion, Multi-source RS image, Genetic-iterative self-organizing data analysis algorithm (GSDA), RS image fusion, Self-adaptive fusion rules, The effect evaluation of the fused image

## Abstract

In order to solve the issue which the fusion rules cannot be self-adaptively adjusted by using available fusion methods according to the subsequent processing requirements of Remote Sensing (RS) image, this paper puts forward GSDA (genetic-iterative self-organizing data analysis algorithm) by integrating the merit of genetic arithmetic together with the advantage of iterative self-organizing data analysis algorithm for multi-source RS image fusion.

The proposed algorithm considers the wavelet transform of the translation invariance as the model operator, also regards the contrast pyramid conversion as the observed operator. The algorithm then designs the objective function by taking use of the weighted sum of evaluation indices, and optimizes the objective function by employing GSDA so as to get a higher resolution of RS image.

As discussed above, the bullet points of the text are summarized as follows.

• The contribution proposes the iterative self-organizing data analysis algorithm for multi-source RS image fusion.
• This article presents GSDA algorithm for the self-adaptively adjustment of the fusion rules.
• This text comes up with the model operator and the observed operator as the fusion scheme of RS image based on GSDA.

The proposed algorithm opens up a novel algorithmic pathway for multi-source RS image fusion by means of GSDA.

## Method details

Since the imaging mechanism and imaging bands of different RS sensor are diverse, so RS image of the same scene, which is generated by disparate RS sensor, there exist redundancy and mutual complementarity of information in multiple RS images. The advantages of RS image generated with disparate sensor can be integrated effectively and smoothly into one RS image, so that more precise and more comprehensive and more reliable image description of identical RS scene can be achieved, and this manipulation can be also realized by subsequent process [1]. The multiple images of the same RS scene, which is generated by unlike RS sensor or diverse imaging manner for the same RS scene, are integrated smoothly into one RS image by using a mathematical model [4].

The typical methods for RS image fusion include IHS (Intensity-Hue-Saturation) transform based on colour space conversion, YUV (YCrCb) transform, principal component analysis transform based on the statistics, Brovey transform, pyramid decomposing transform based on multi-scale analysis [5], DWT transform (Discrete Wavelet Transform), Contourlet transform [6], DAA (Data Assimilation Arithmetic), and GPSA (Genetic-Particle Swarm Algorithm), etc. In the above fusion approaches RS image fusion, the fusion image based on HIS is obviously insufficient in spectral preservation due to colour distortion [7]. Similarly, the fused image based on YUV is though conspicuous in details and characteristics, but spectral distortion of the fused image is extremely serious. Besides, along with the increase of the decomposition layers of DWT transform, the fused image based on DWT is though abundant in detailed information, but spectral retentivity of the fused image is slightly worse [9]. In addition, the image fused with DAA is although moderately obvious in spatial details, but spectral distortion of the fused image is likewise marginally serious [10]. In like manner, the image fused with GPSA though possesses better spectral retention, but texture information of the fused image is slightly ambiguous, and image clarity gradually decreases along with the increase of the iterations [11]. Similarly, although the image fused with combined DAA and GPSA retains more detail information of the image, but spectral torsion resistance of the fused image will become more and more serious along with the increase of decomposition layers of the image [12].

In spite of the fact that the above methods have achieved with some successes, but their fusion rules must be determined before the inosculation of RS image, and these fusion rules cannot be adjusted self-adaptively [3]. Moreover, it is also problematic for the fusion of RS image to synthesize the respective virtues of these methods [2]. In order to solve the above problems, the iterative optimization algorithm GSDA is presented for the integration of RS image in this article [8]. The proposed GSDA can be further used to dispel the spectral distortion of the fused image brought by DAA and GPSA, and the texture information of the fused image will become clearer. The proposed GSDA can be also used especially to eliminate the spectral torsion resistance introduced by DAA and GPSA, and the clarity of the fused image will become ever more perfect [13]. For this purpose, the observed data are efficiently combined with simulated data generated with GSDA so as to achieve the fused RS image that is more objective and closer to natural inosculation [2]. The proposed algorithm of RS image fusion can be used to self-adaptively modulate the fusion rule of RS image by combining with the merits of different fusion methods according to the subsequent requirement of RS image processing [14]. Thus, the proposed scheme of RS image fusion solves the urgent issues that the fusion rules of RS image cannot be self-adaptively modulated by using traditional fusion approaches [15]. In order to verify the effectiveness and superiority of the proposed algorithm, some of the instances of RS image fusion are presented in this experiment [18].

## The optimized algorithm of GSD

**Fig. 1:**
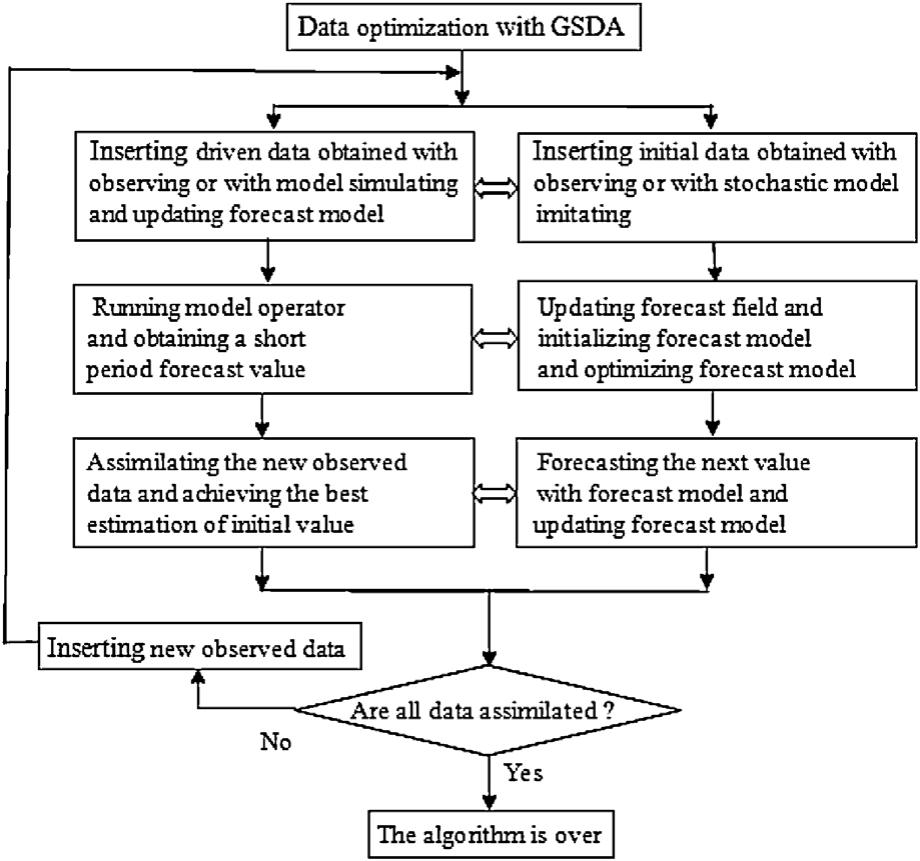
The algorithmic flow chart of data optimization with GSDA.

The essence of GSDA is that the observed data are effectively combined with the simulation data by means of a mathematical mode, as a result, an analytical result of RS image data which is more objective and closer to the natural scene is achieved in the end [16]. The algorithmic flow chart of data optimization with GSDA is shown in Fig. 1. The algorithmic procedure of GSDA is summarized as follows.

(1) The forecast field of the model is regarded as an initial estimated field.
(2) The updated field is initialized.
(3) Several steps of the forward-forecast are performed with the model, and then the recent forecast field is regarded as the next updated initial estimation field.
(4) If all data are optimized, then the algorithm is over. Otherwise the algorithm returns to step (1) and continues to iterate.

This iteration is repeated several times, so as to come into a cyclic process as follows: Inserting observed data into the system → Updating forecast field → Initializing the system → Forecasting estimated data with the model → Inserting observed data into the system → Updating forecast field → Initializing the system → Forecasting estimated data with the model → … End. At the beginning of each circulation in the process of optimization, the system continuously updates the forecast field by the aid of recent observed data. The system is updated with two dissimilar methods: (1) the observed data that are weighted with the mean value of several data are directly substituted for the forecast values that are the nearest grid point value of the model; (2) taking the forecast field as an initial estimated field, the observed data are objectively analyzed. The former pattern is known as the direct insertion of the observed value, or the immediate update of the forecast value, that is, one-dimensional optimization of the observed data. The latter pattern is called the indirect insertion of the observed value, or the indirect update of the forecast value, that is, two-dimensional optimization of the observed data.

GSDA consists generally of the model operator, the observed operator, the objective function, and the optimization algorithm, etc. Taking the optimized objective function as a destination, the objective function *F*(*y*(*τ*_*j*_)) is defined as follows.

(1) $$F(y(\tau_{j}))=\frac{1}{2}{\left[y(\tau_{j})-y^{\prime }(\tau_{j})\right]}^{T}C^{-1}[y(\tau_{j})-y^{\prime }(\tau_{j})]+\frac{1}{2}{\sum_{j=0}^{M}\left[G_{j}(L(s_{j}))-{x^{\prime }}_{j}\right]}^{T}D_{j}^{-1}[G_{j}(L(s_{j}))-{x^{\prime }}_{j}]$$

where *F*(*y*(*τ*_*j*_)) is the objective function; *τ*_*j*_ denotes the moment *j*; *y*(*τ*_*j*_) is the initial value of the state vector, and it is also the column matrix of the optimized variable, and it denotes the initial state of assimilation period *j*; *y*′*τ*_*j*_ is the ambient field; *C* is the covariance matrix of the error of the ambient field; ${x^{\prime }}_{j}$ is the observed value of the moment *j*, and it is multi-dimensional vector of the observed data; *L* is the model operator; *s*_*j*_ is the *j*th observed parameter of *L*; *G*_*j*_ is the observed operator; *D*_*j*_ is the covariance matrix of the error of the observation field.

### The wavelet transform of the translation invariance

Since RS image fusion with the wavelet transform-based can be used to decompose RS image into several distinct frequency bands, so the different fusion method can be adopted in the diverse frequency bands. As a result, distinguishing features of the original RS image in different frequency domain are reserved in the composite image [17]. The original RS image can therefore be considered as driven data of the present moment, and the fusion pattern of RS image based on the wavelet transform of the translation invariance can be regarded as the model operator. The model operator of GSDA can be used for short-term forecast of the next moment by using the driven data of the present moment in geoscience research domain. The basic procedure of GSDA for RS image fusion is defined as follows.

(1) The original RS image is geometrically accurately rectified.
(2) The original RS image is processed by using the wavelet transform of the translation invariance, so as to generate the low-frequency and the high-frequency component coefficients of the wavelet transform for diverse resolution and different direction.
(3) The low-frequency component coefficients of the wavelet transform are fused with the weighted-mean method, and then the high-frequency component coefficients of the wavelet transform are fused with the maximum operator model.
(4) The image that is generated with the inverse wavelet transform is regarded as the forecast image.

### Contrast pyramid decomposition

RS image fusion model based on the contrast pyramid decomposition is regarded as the observed operator in this experiment. The observed operator of GSDA is used to generate the observed value of diverse moments, and then the fused image generated with the model is considered as the observed data of GSDA. The above algorithm is defined as below.

(1) Contrast pyramid decomposition is respectively performed for each original RS image, as a result, the contrast pyramids for each original image are respectively established.
(2) Each decomposed layer of the image pyramid is respectively syncretized. Then the low-frequency components of the decomposed image are processed with the weighted-mean operator, and the high-frequency components of the decomposed image are processed with the maximum operator. As a result, the contrast pyramid of the fused image is generated in the end.
(3) The contrast pyramid of the fused image is decomposed with the inverse contrast pyramid transform, and then the rebuilt image that is generated by the above algorithm is exactly the fused RS image.

### Genetic-iterative algorithm of DSDA

GSDA is taken as an optimization algorithm for RS image fusion in this experiment. GSDA is specifically described as follows.

Suppose that *V*_*m*_ is m-dimensional search space of the objective (*m* is number of optimization variable), then there exists the population *ξ* = {*ξ*_1_, *ξ*_2_, … *ξ*_*n*_} that consists of *n* particles. The position of the particle *j*, *ξ*_*j*_ = {*ξ*_*j*1_, *ξ*_*j*2_, … *ξ*_*jm*_}, indicates the *j*th solution of the algorithm. Each particle searches the latest algorithmic solution by continually adjusting its own position *ξ*_*j*_. The optimal solution (fitness degree of particle), which is searched by the particle *j*, is marked as *q*_*js*_, and the optimal solution (colony fitness degree), which is searched at present by the whole particle swarm, is marked as *q*_*ks*_. The velocity of the particle *j* in the particle population is marked as *U*_*j*_ = {*u*_*j*1_, *u*_*j*2_, … *u*_*jm*_}. When both the two optimal solution *q*_*js*_ and *q*_*ks*_ are found out, then the particle updates its peculiar velocity with formula (2), and then the particle adjusts its individual position with formula (3).

(2) $$u_{js}(\tau +1)=wu(\tau )+\alpha \text{rand}()(q_{js}-\xi_{js}(\tau ))+\beta \text{rand}()(q_{ks}-\xi_{js}(\tau ))$$

(3) $$\xi_{js}(\tau +1)=\xi_{js}(\tau )+u_{js}(\tau +1)$$

where *u*_*js*_(*τ* + 1) is the s-dimensional velocity of the particle *j* in the *τ* + 1 iteration; *ξ*_*js*_(*τ* + 1) is the s-dimensional position of the particle *j* in the *τ* + 1 iteration; *w* is an inertia weighted coefficient; *α* and *β* are the accelerated parameter determined by the iteration, respectively; Function rand()∈(0,1) indicates the random number between (0,1). The iteration optimized algorithm of GSDA can be expounded as follows.

(1) Both the initial position and initial velocity of each particle in the particle population are respectively initialized in search space.
(2) Both the update velocity and update position of each particle are calculated respectively with formula (2) and (3).
(3) Particle fitness degree *q*_*js*_ of the particle *j* is calculated (*j* = 1, 2, …, *n*), and colony fitness degree *q*_*ks*_ of the particle *j* is likewise calculated (*k* = 1, 2, …, *l*).
(4) For the particle *j* (*j* = 1, 2, …, *n*), if its current fitness degree is better than the fitness degree *q*_*js*_ of its best position it has experienced, then the value of its current fitness degree is assigned to *q*_*js*_.
(5) For particle *j*, both its particle fitness degree *q*_*js*_ (*j* = 1, 2, …, *n*) and its colony fitness degree *q*_*ks*_ (*k* = 1, 2, …, *l*) are respectively compared with the current *q*_*js*_ and *q*_*ks*_. If there exist better *q*_*js*_ and *q*_*ks*_, then both *q*_*js*_ and *q*_*ks*_ at present are respectively updated with better *q*_*js*_ and *q*_*ks*_.
(6) The value of fitness degree of each particle is ranked ordering from greater to smaller order. For posterior particles in the sequence, the following interlacing and variation operation are performed, respectively, and then the original particle is replaced with a newly generated particle.
(7) The crossover operation is run. The particles of the parent population are randomly mated processing. It is haphazard to determine the crossing position for each pair of particles of the parent population, and then the latest particles of the descendant population are generated by the interlacing operation.
(8) The variation operation is carried out. A number j∈{1,2,…,m} is selected randomly and discretionarily. Then the *j*th variable *ξ*_*ij*_ of the particle *ξ*_*i*_, whose variation is going to be generated, executes the variation operation with formula (4).

(4) $${\xi^{\prime }}_{ij}=\xi_{ij}+\text{ran}{\text{d}}^{\prime }()(\xi_{jv}-\xi_{js})$$

where function $\text{ran}{\text{d}}^{\prime }()\in (-1,1)$ shows the random number between (−1,1); *ξ*_*jv*_ is the value of fitness degree of the particle *j* before the variation; *ξ*_*js*_ is the value of colony fitness degree of the particle *j* before the variation. Both the update velocity and update position of each particle are respectively calculated with formulae (2), (3).

(9) If the update velocity and update position of each particle are all optimal, then the algorithm is over. Otherwise, the operation returns to step (2) and continues to iterate.

**Fig. 2:**
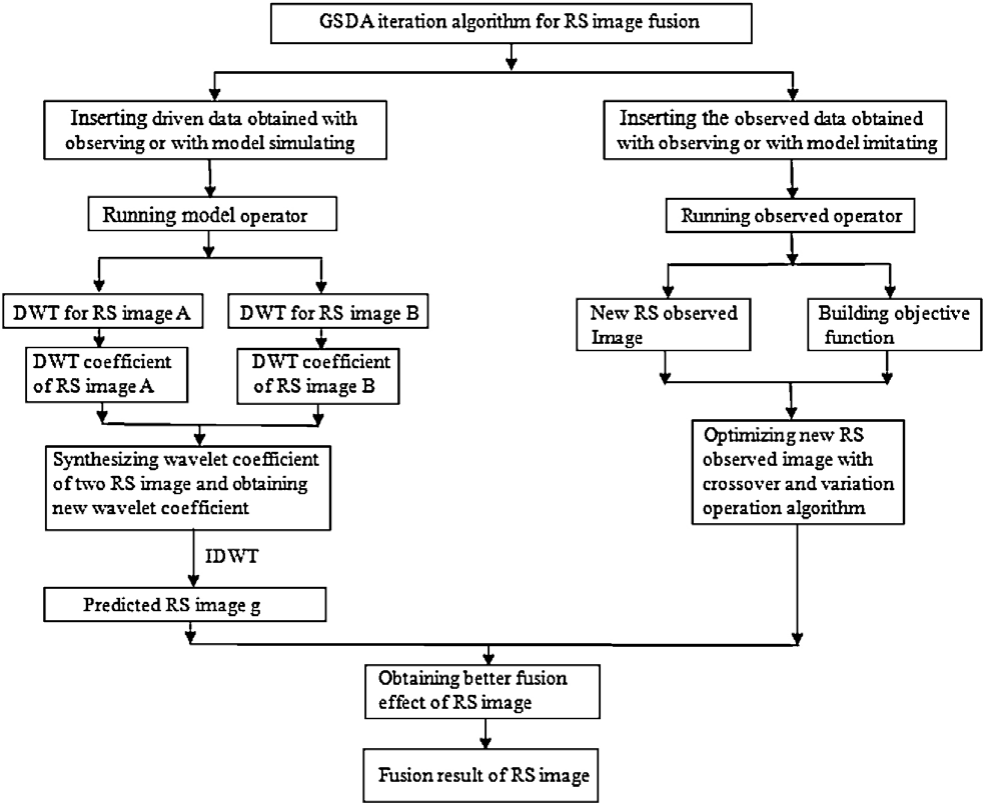
The algorithmic flow chart of the GSDA iteration procedure.

The algorithmic flow chart of GSDA iteration procedure is shown in Fig. 2.

## RS Image fusion based on the optimal GSDA

In this experiment, the original RS image data are considered as the driven data of GSDA, and RS image data that are generated with the wavelet transform method (the model operator) are regarded as the predicted data, and then RS image data that are generated with the contrast pyramid transform approach (the observed operator) are regarded as the observed data [19]. Afterwards, the weight vector of each attribute index is determined according to the influence extent of the subsequent processing, which is generated with each attribute index of the fused image. Then the weighted sum of the multi-evaluation indices of the fused image is considered as the objective function of GSDA. After that, the objective function is optimized with the optimization algorithm of GSDA, and the solution of the objective function is regarded as the final result image. Flow chart of the above algorithm is shown in Fig. 2. The principle of the above algorithm is summarized as follows [21].

(1) The model operator is run. The wavelet decomposing level of the wavelet transform of translation invariance is divided respectively into 1–4 layers. Then the image that is generated with the wavelet decomposition is considered as the forecast data of GSDA.
(2) The observed operator is performed. The decomposing level of the contrast pyramid transform is divided respectively into 3–6 layers. Then the image that is generated with the contrast pyramid transform is regarded as the observed data of GSDA.
(3) The objective function of GSDA is built. The evaluation index of the fused image, such as the standard deviation, mean gradient, information entropy, and the spatial frequency, is respectively regarded as the corresponding objective function in this experiment.
(4) The optimization algorithm is initialized. The fused image that is generated by steps (1) and (2) is considered as the initial particle swarm, and the value of the objective function is considered as the adaptive-degree value of the particle. Then termination rules of the iteration are established. That is, maximum iterations are up to 100; or the adaptive-degree of the global optimal particle generated in ten continuous iterations is no longer progressive.
(5) The optimization algorithm is carried out. In other words, the objective function is optimized with GSDA, and the final fused RS image is generated as the result image.

## Experiment of multi-source RS image fusion

### Optimized indexes for fusion effect evaluations of RS image

The following indexes are introduced for fusion effect evaluation of multi-source RS image in this experiment.

#### Degree of clarity (DC)

Degree of clarity (DC) is used to measure the clear extent of the fused RS image, and it reflects the contrast of minute details and the changing characteristics of the texture in the image. The greater the change rate of the grey level is, the greater the gradient will be, and so the higher the clear degree of RS image will be. Degree of clarity (DC) for RS image fusion is defined as follows.

(5) $$\text{DC}=\frac{1}{\left(M-1)(N-1\right)}{\sum_{x=0}^{M-1}\sum_{y=0}^{N-1}\left\{\frac{1}{2}[{\left(f(x,y)-f(x,y+1)\right)}^{2}]+[{\left(f(x,y)-f(x+1,y)\right)}^{2}]\right\}}^{1/2}$$

where DC is the degree of clarity of the image *f*; *f*(*x*, *y*) is the pixel value of the image *f* at point (*x*, *y*), *x* = 0, 1, 2, …, *M* − 1, *y* = 0, 1, 2, …, *N* − 1; *M* is the line number of the image *f*; *N* is the column number of the image *f*. The meaning of the same symbol below is as the same as stated above.

#### Information entropy (IE)

The increase of the amount of information is a fundamental requirement of RS image fusion, and it can be used to reflect by changes in information entropy (IE) before and after the RS image is integrated. Information entropy (IE) of the image is the average information amount of the image, and information entropy (IE) is defined as follows.

(6) $$\text{IE}=-\sum_{i=0}^{L}P(i)\;\log_{2}\;(P(i))$$

where IE is information entropy of the image *f*; *P*(*i*) is the present probability density of the grey value of the pixel *i*; *L* is the ranges of the grey value of the pixel *i*, and generally *L* = 0–255.

#### Cross entropy of root mean square (CE)

Cross entropy of root mean square (CE) of two images is used to measure the information difference between the two images. Furthermore, the smaller the CE is, the less the information difference between the two images will be. Contrarily, the greater the CE is, the richer the information difference between the two images will be. Cross entropy of root mean square (CE) for the original image *A* and *B* with the fused image *F* is defined as follows.

(7) $$\text{CE}=\sqrt{\frac{\text{C}{\text{E}}_{A,F}^{2}+\text{C}{\text{E}}_{B,F}^{2}}{2}}$$

(8) $${\text{CE}}_{A,F}=\sum_{i=0}^{L-1}P_{A_{i}}\;\log_{2}\frac{P_{A_{i}}}{P_{F_{i}}}$$

(9) $$CE_{B,F}=\sum_{i=0}^{L-1}P_{B_{i}}\log_{2}\frac{P_{B_{i}}}{P_{F_{i}}}$$

where CE is the cross entropy of root mean square for the original image *A* and *B* with the fused image *F*; CE_*A*,*F*_ and CE_*B*,*F*_ are the cross entropy of the original image *A* and *B* with the fused image *F*, respectively; *P*_*A*_, *P*_*B*_, and *P*_*F*_ are the joint probability density of the image *A*, *B* and the fused image *F*, respectively.

#### Mean value $\bar{f}$

Mean value $\bar{f}$ is used to express the average value of the pixel grey-level of the image. Mean value $\bar{f}$ is defined as follows.

(10) $$\bar{f}=\frac{1}{M\times N}\sum_{x=0}^{M-1}\sum_{y=0}^{N-1}f(x,y)$$

where $\bar{f}$ is the mean value of the image *f*. The meaning of the other symbol is the same as stated above.

#### Standard deviation (SD)

Standard deviation (SD) is used to reflect the distributing situation of the pixels of the image. Standard deviation (SD) is defined as follows.

(11) $$\text{SD}=\sqrt{\frac{1}{M\times N}\sum_{x=0}^{M-1}\sum_{y=0}^{N-1}{\left(f(x,y)-\bar{f}\right)}^{2}}$$

where SD is the standard deviation of the image *f*. The meaning of the other symbol is the same as stated above.

#### Root-mean-square error (RMSE)

Root-mean-square error (RMSE) is used to measure the error of root mean square between the original image and the fused image. Furthermore, the less the RMSE, the more consistent the original image and the fused image will be. Root-mean-square error (RMSE) is defined as follows.

(12) $$\text{RMSE}=\sqrt{\frac{1}{M\times N}\sum_{x=1}^{M}\sum_{y=1}^{N}{\left(R(x,y)-F(x,y)\right)}^{2}}$$

where RMSE is the root-mean-square error; *R*(*x*, *y*) is the original image; *F*(*x*, *y*) is the fused image. The meaning of the other symbol is the same as stated above.

#### Mean gradient (MG)

Mean gradient (MG) is used to measure the clear extent, the detailed contrast of the image, and the characteristics of the texture transform of the image, etc. Mean gradient (MG) is defined as follows.

(13) $$\text{MG}=\frac{1}{M\times N}\sum_{x=0}^{M-1}\sum_{y=0}^{N-1}\sqrt{\frac{\Delta I_{x}^{2}+\Delta I_{y}^{2}}{2}}$$

where MG is the mean gradient of the image; Δ*I*_*x*_ and Δ*I*_*y*_ are the first order difference of the pixel (*x*, *y*) in *x* and *y* direction, respectively. The meaning of the other symbol is the same as stated above.

#### Spatial frequency (SF)

Spatial frequency (SF) is used to reflect the overall activity extent in the spatial domain of the image. Spatial frequency (SF) is divided into the line frequency RF and the column frequency CF. Line frequency RF of the image is defined as follows.

(14) $$\text{RF}=\sqrt{\frac{1}{MN}\sum_{x=0}^{M-1}\sum_{y=1}^{N-1}{\left[f(x,y)-f(x,y-1)\right]}^{2}}$$

Column frequency CF of the image is defined as follows.

(15) $$\text{CF}=\sqrt{\frac{1}{MN}\sum_{x=0}^{N-1}\sum_{y=0}^{M-1}{\left[f(x,y)-f(x-1,y)\right]}^{2}}$$

Spatial frequency SF of the image is defined as follows.

(16) $$\text{SF}=\sqrt{{\text{RF}}^{2}+{\text{CF}}^{2}}$$

where RF is the line frequency of the image; CF is the column frequency of the image; SF is the spatial frequency of the image. The meaning of the other symbol is the same as stated above.

#### Correlation coefficient (CC)

Correlation coefficient (CC) is used to describe the correlation degree between the original image and the fused image. Correlation coefficient (CC) between the original image and the fused image is defined as follows.

(17) $$\text{CC}=\frac{\sum_{x=1}^{M}\sum_{y=1}^{N}\left[F(x,y)-\bar{F}(x,y)][R(x,y)-\bar{R}(x,y)\right]}{\sqrt{\sum_{x=1}^{M}\sum_{y=1}^{N}{\left[F(x,y)-\bar{F}(x,y)\right]}^{2}\sum_{i=1}^{M}\sum_{j=1}^{N}{\left[R(x,y)-\bar{R}(x,y)\right]}^{2}}}$$

where CC is the correlation coefficient between the original image and the fused image; *R*(*x*, *y*) is the grey-level value of the original image at pixel point (*x*, *y*); $\bar{R}(x,y)$ is the average grey-level value of *R*(*x*, *y*); *F*(*x*, *y*) is the grey-level value of the fused image at pixel point (*x*, *y*); $\bar{F}(x,y)$ is the average grey-level value of *F*(*x*, *y*). The meaning of the other symbol is the same as stated above.

#### Signal-to-noise ratio (SNR)

Signal-to-noise ratio (SNR) is used to reflect the energy ratio between the original image and the fused image. Furthermore, the higher the signal-to-noise ratio is, the better the quality of the fused image will be. Signal-to-noise ratio (SNR) is defined as follows.

(18) $$\text{SNR}=10\;\log\frac{\sum_{x=1}^{M}\sum_{y=1}^{N}F^{2}(x,y)}{\sum_{x=1}^{M}\sum_{y=1}^{N}{\left[R(x,y)-F(x,y)\right]}^{2}}$$

where SNR is signal-to-noise ratio; *R*(*x*, *y*) is the grey-level value of the original image at pixel point (*x*, *y*); *F*(*x*, *y*) is the grey-level value of the fused image at pixel point (*x*, *y*). The meaning of the other symbol is the same as stated above.

#### Peak-Signal-to-Noise Ratio (PSNR)

Peak-Signal-to-Noise Ratio (PSNR) is used to represent the peak energy ratio between the original image and the fused image. Furthermore, the higher the peak-signal-to-noise ratio is, the better the effect of the fused image will be. Peak-Signal-to-Noise Ratio (PSNR) is defined as follows.

(19) $$\text{PSNR}=10\;\log\frac{{\left(L-1\right)}^{2}}{{\text{RMSE}}^{2}}$$

Where, PSNR is the peak-signal-to-noise ratio; *L* is the number of the grey-level of the image; RMSE is the root mean square error. The meaning of the other symbol is the same as stated above.

### Fusion experiments of orthopanchromatic aerial RS image and multi-spectral space RS image

**Fig. 3:**
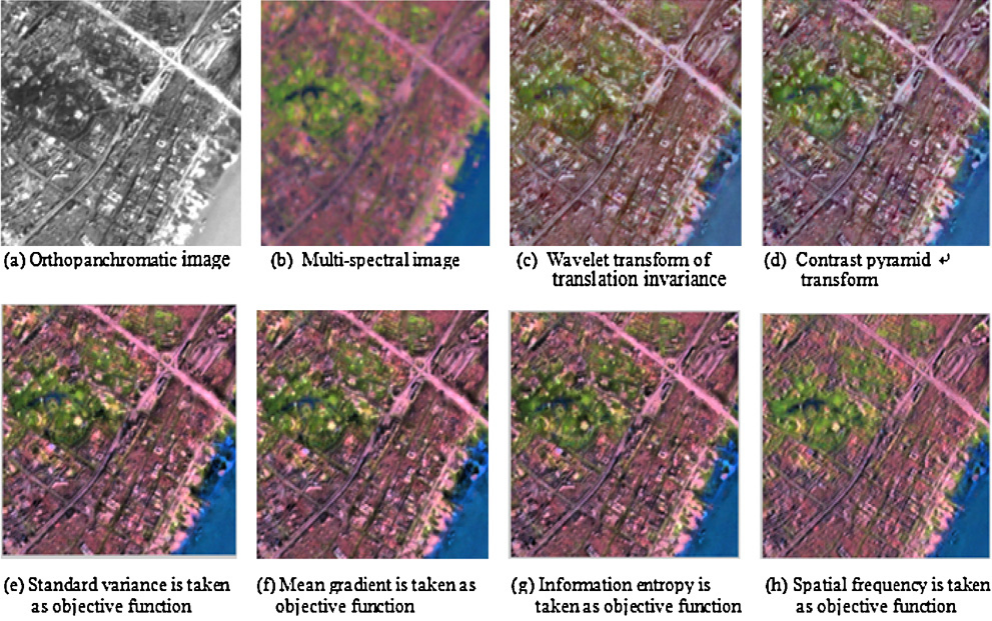
Comparison of several different fusion methods for panchromatic RS image and multi-spectral RS image.

Fused RS images by using above various methods are shown in Fig. 3. It is visually evident that RS image fused with the proposed algorithm effectively improves the spatial resolution of RS image while it retains spectral information of multi-spectral space RS image (see Fig. 3(c)–(h)). The quantitative indices are introduced in the experiment for assessing the quality of the fused RS image [22]. These evaluation indices of the fused image include a series of parameter index, such as standard deviation (SD), mean gradient (MG), information entropy (IE), cross entropy of root mean square (CE), spatial frequency (SF), structural comparability (SC), etc. Where, the standard deviation is used to determine the divergence which the grey-level value of the image is relative to the mean value, and the greater its value, then the greater the variation range of the grey-level of the image, and the greater the contrast of the image, also the more abundant the amount of information of the image, and the higher the resolution of the image as well. The mean gradient is utilized to reflect the contrast and textural characteristics of slight details in RS image. Generally speaking, the greater its value is, the clearer the RS image will be. The information entropy is applicable to reflect the amount of information of RS image, and the greater its value, the more plentiful the information recorded in RS image. The spatial frequency is used to reflect the overall activity level of the image space. Structural comparability (SC) of the image is used to reflect the similarity extent of two images, and it is also an objective evaluation standard for the quantitative assessment of the fused image [23]. In addition, SC is also a synthetical assessment operator for the quantitative evaluation of the fused image in the brightness, contrast and structure of the image [24]. Meanwhile, SC is synthetically considered in three fields, that is, the light intensity, contrast, and structure of the image [25].

Comparison of the quantitative index data of the fused image in this experiment is given in Table 1. In Table 1, the wavelet transform of the translation invariance is used to decompose RS image into three layers, and the contrast pyramid transform is likewise used to decompose RS image into three layers as well. Besides, the standard deviation (SD), mean gradient (MG), information entropy (IE) spatial frequency (SF), and structural comparability (SC) are respectively regarded as the objective function for RS image fusion under GSDA scheme. Moreover, variable R, G, and B show red band, green band, and blue band of RS image, respectively.

It can be seen from Table 1 that the index data of the evaluation with the standard deviation, mean gradient, information entropy and spatial frequency, which reflect the enhancement of information in spatial details of RS image, are greatly improved than those with the wavelet transform of the translation invariance, and obviously ameliorated than those with the contrast pyramid transform as well. Moreover, the consequence of visual interpretation of RS image indicates that spectral retentivity of various algorithms is commensurate to each other, and spectral information of the original RS image is preferably retained.

### Fusion experiment of high-resolution aerial RS image and multi-spectral spatial RS image

**Fig. 4:**
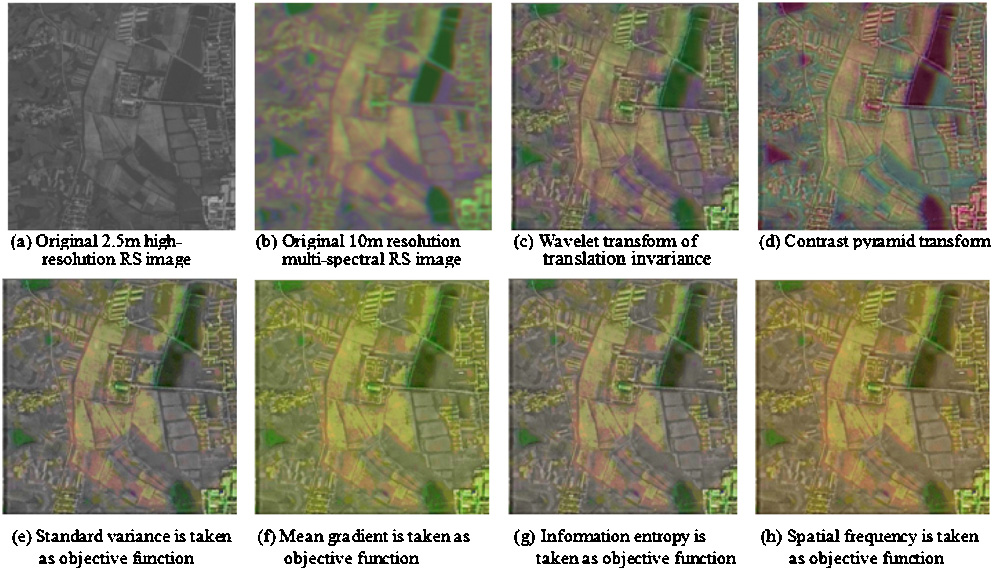
Comparison of several different fusion methods for high-resolution RS image and multi-spectral RS image.

Since the orthopanchromatic sensor is highly sensitive to the bright target, so it possesses a better detective property for the brightness target, but it is insensitive to the colour change of RS scene. Therefore, the image of the road is clearly visible in the panchromatic RS image shown in Fig. 3(a), but the images of the buildings, grassland, and other background characteristics are indistinctly and obscure. Contrariwise, for the multi-spectral spatial RS image of the same scene, since the light is dim and murky, so the similar screenage of the image is almost illegible. However, the backgrounds of the same targets and other ground objects in the fused image, such as water bodies, meadows, houses, and others, are yet visible vaguely (see Fig. 3(c)–(h)). The original panchromatic aerial high-resolution RS image and the multi-spectral space RS image as well as the fused result image that is generated with six methods mentioned above are shown in Fig. 4. It can be markedly seen from Fig. 4, from the whole effect of manual visual judgement, that the distinct images of the panoramas are all generated by using six algorithms. Moreover, the images of the main ground objects are also clear and distinguishable, such as farmlands, roads, buildings, meadow lands, aqueduct, terraced fields, and other ground objects (see Fig. 4(c)–(h)).

In this experiment, the comparisons of the quantitative indices of the fused image are given in Table 2. In Table 2, the wavelet transform of the translation invariance is used to decompose RS image into three layers, and the contrast pyramid transform is likewise utilized to decompose RS image into three layers as well. Furthermore, the standard deviation, mean gradient, information entropy, and spatial frequency are respectively alone regarded as the objective function of RS image fusion under GSDA frame. Where, variable SD, MG, IE, SF, and CE respectively indicate the standard deviation, mean gradient, information entropy, spatial frequency, and the cross entropy of root mean square between the original image and fused image (see Table 2).

It can be further seen from Table 2 that the index data, such as standard deviation, mean gradient, information entropy, and the spatial frequency, which respectively reflects the texture information of the spatial details of RS image, are all improved greatly with the proposed algorithm than those with the wavelet transform of the translation invariance and observably ameliorated than those with the contrast pyramid transform. Moreover, it is also conformable with the effect of the visual interpretation.

### Comparative analysis of different fusion method of RS image

**Fig. 5:**
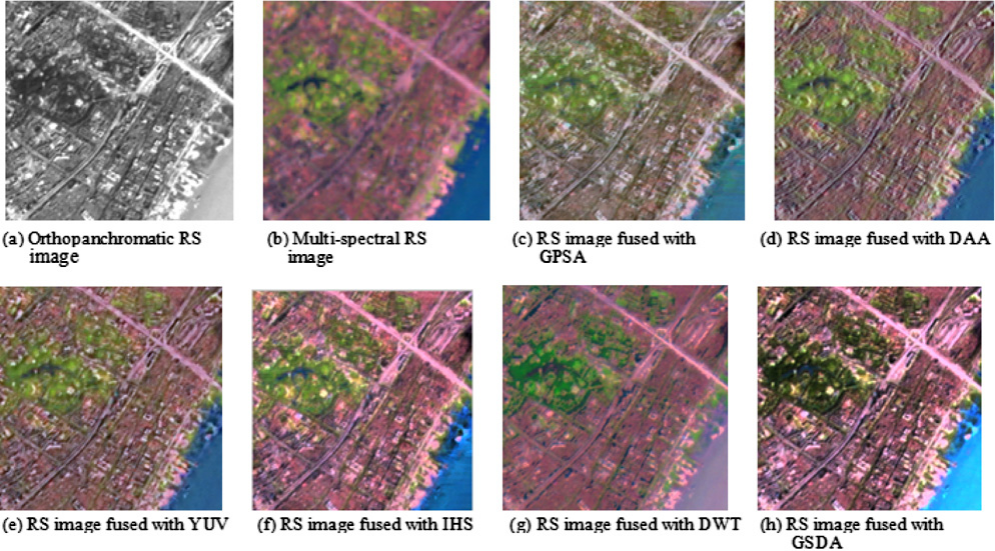
Comparison of several different fusion methods for RS image amalgamation.

Comparisons of several different fusion methods for the amalgamation of RS image are shown in Fig. 5. (a)–(h) is respectively an orthopanchromatic RS image, a multi-spectral RS image, a RS image fused with GPSA, a RS image fused with DAA, a RS image fused with YUV, a RS image fused with IHS, a RS image fused with DWT, and a RS image fused with GSDA. It can be evidently seen from Fig. 5(c) that, from the visual interpretation effect, the image fused with GPSA is although provided with slightly better spectral retention, but the details of the image is imperceptibly ambiguous and vague (Fig. 5(c)). It can be also obviously seen from Fig. 5(d) that, from the visual evaluation result, the image fused with DAA is although more prominent in spatial details, but the fused image is very insufficient in the spectral retention of (see Fig. 5(d)). It can be similarly clearly seen from Fig. 5(e) that, from the whole point of view of the spectral distribution, the image fused with YUV is though obvious in the details and characteristics, but the spectral distortion of the fused image is extremely serious. It can be further distinctly seen from Fig. 5(f) that, from the point of the effect of the optical interpretation, the fusion algorithm of RS image based on IHS is manifestly superior to the arithmetic based on YUV space. The details of the image features as well as the characteristics of ground objects are clearly discernible (see Fig. 5(f)). However, the algorithm of RS image fusion based on IHS is manifestly insufficient in the spectral preservation compared to that of the arithmetic based on YUV space. Although the algorithm mentioned above is very superior to that shown in Fig. 5(f) and (g) in the spectral preservation and the spatial detail information, but the fusion effect is slightly worse compared to that of the algorithm based on GSDA shown in Fig. 5(h). It can be further markedly seen from Fig. 5(h) that, from the overall effect of manual visual judgement, the fusion algorithm of RS image based on GSDA is signally superior to the other algorithms mentioned above. The image fused with the proposed algorithm is not only provided with the significant spectral preservation, but also possesses the space detail information of the image features as well as the characteristic information of ground objects. Furthermore, the details of the image features as well as the spectral characteristics of ground objects are obviously recognizable (see Fig. 5(h)).

The above analysis naturally leads us to the following possible conclusion that, compared to the traditional integration algorithms, such as GPSA, DAA, YUV, IHS, DWT, and the like, the proposed GSDA is very effective and excellent in the application of RS image fusion. Comparisons of different fusion methods for orthopanchromatic RS image and multi-spectral RS image are given in Table 3. It can be seen from Table 3 that the index data, such as SD, MG, IE, SF, and CE, which respectively reflects the spatial detail information of the fused image, are all improved greatly with the proposed algorithm, and superior to those with GPSA, DAA, YUV, IHS, and DWT. Considering the possible reasons, it is rational for us to conclude that the proposed GSDA is relatively optimal compared to the traditional algorithm, such as the six algorithms mentioned above (see Table 3).

### Analyses and discussions

It can be clearly seen from Table 1, Table 2, for the qualitative evaluation of the fused RS image, that the proposed algorithm is much better than that of the wavelet transform of the translation invariance, and also more superior to that of the contrast pyramid transform in accordance with the cross entropy of root mean square as well. This indicates that GSDA can be used to perfectly optimize the objective function without introducing any incorrect information. In addition, it can be further seen from Table 1, Table 2 that it is a fundamental issue to build an appropriate objective function for the integration of RS image. Generally speaking, the superiority or inferiority of the objective function directly concerns the ultimate fusion effect of RS image [17]. In this way, the spectral information of the original multi-spectral RS image can be perfectly preserved in the fused image generated with the transformation of the contourlet-based. Furthermore, the texture information of the original panchromatic RS image can be distinctly preserved in the fused image generated with the transformation of the principal component analysis-based, etc. Therefore, it is radically important for selecting the optimal approach to build the model operator and the observed operator according to specific applied purpose in the practical applications.

## Conclusions

Experimental results demonstrate that the proposed scheme of RS image fusion is extremely effective and superior to that of the traditional scheme, such as the six algorithms discussed previously. The important conclusion to be drawn from this discussion is that the proposed algorithm solved the problem which the fusion rules cannot be self-adaptively adjusted by using traditional fusion ways according to the subsequent processing requirements of RS image.

All mentioned above tell us that it is of great importance for building an appropriate objective function to smoothly syncretize the multi-spectral image and the panchromatic image into an integrated RS image. However, one thing we have to notice is that it is considerably important for choosing an optimal method to build the model operator and the observed operator in terms of a certain applied purpose in practical RS image fusion. Considering the possible reasons, it is rational for us to conclude that the proposed GSDA is relatively optimal in RS image fusion. In a word, the proposed GSDA of RS image fusion carries forward the benefits of traditional fusion ways, such as IHS, YUV, principal component analysis transform, Brovey transform, pyramid decomposing transform, DWT, Contourlet transform, DAA, and GPSA, etc., and introduces the model operator and observed operator so as to improve the quality of the fused image. Experimental results show that the proposed GSDA is much effective and the most optimal compared to the conventional fusion method for RS image amalgamation. There is no doubt, as discussed above, that the proposed algorithm opens up a novel algorithmic pathway for RS image fusion by means of GSDA.

## Figures and Tables

**Table 1:** Comparison of different fusion methods for orthopanchromatic RS image and multi-spectral RS image.

Fusion methods	Band	SD	MG	IE	SF	SC
Wavelet transform of translation invariance	R	47.8652	16.4362	7.6458	25.4526	0.7256
	G	47.7246	16.2436	7.4672	25.5281	0.7163
	B	47.8138	16.3274	7.5237	25.5134	0.6581
Contrast pyramid transform	R	48.2649	18.2541	7.8425	27.3542	0.7563
	G	47.3478	17.4857	7.6453	26.3275	0.7428
	B	48.3283	17.8542	7.5728	27.3428	0.7553
Standard deviation is taken as objective function	R	54.6318	18.2448	7.6468	28.4653	0.7642
	G	55.7326	17.6237	7.7482	28.5367	0.7865
	B	56.3249	17.8456	7.8365	28.8396	0.7754
Mean gradient is taken as objective function	R	57.7652	17.6568	7.9436	28.7904	0.6543
	G	57.7269	18.2485	7.8945	28.3576	0.6756
	B	57.6954	18.3547	7.9256	28.4859	0.7243
Information entropy is taken as objective function	R	58.7265	18.4639	7.8542	29.6521	0.8492
	G	58.6495	18.5276	7.8936	29.7854	0.8379
	B	58.7392	18.6495	7.8794	29.8963	0.8543
Spatial frequency is taken as objective function	R	57.3982	17.3967	7.8644	28.3257	0.7659
	G	57.4149	17.4798	7.8926	28.4795	0.7862
	B	57.5218	17.5126	7.9021	28.5243	0.7943

**Table 2:** Comparison of different fusion methods for high-resolution RS image and multi-spectral image.

Fusion methods	Band	SD	MG	IE	SF	CE
Wavelet transform of translation invariance	R	58.6245	18.2536	8.6845	29.3698	2.2154
	G	58.6825	18.2758	8.7096	29.3926	2.2465
	B	58.7562	18.3126	8.6934	29.4269	2.2698
Contrast pyramid transform	R	47.3216	17.3267	7.3542	28.4581	1.8234
	G	47.3638	17.3864	7.3824	28.4832	1.8567
	B	47.4135	17.4125	7.4087	28.5021	1.9026
Standard deviation is taken as objective function	R	56.3641	18.3531	8.6845	29.4215	2.7854
	G	56.3851	18.3742	8.7093	29.4532	2.8025
	B	56.4238	18.4016	8.7269	29.4854	2.8165
Mean gradient is taken as objective function	R	58.6542	17.3674	7.8392	28.3568	1.6845
	G	58.6349	17.3825	7.8768	28.3792	1.6904
	B	58.6843	17.4123	7.9142	28.4123	1.7125
Information entropy is taken as objective function	R	59.3641	18.5436	8.6574	29.6471	2.4357
	G	59.3842	18.5624	8.6896	29.6639	2.4536
	B	59.4068	18.6092	8.7158	29.6945	2.4765
Spatial frequency is taken as objective function	R	57.6543	17.4126	7.8342	28.4231	1.6574
	G	57.6824	17.4537	7.8564	28.4624	1.6892
	B	57.7127	17.4896	7.8968	28.4895	1.7014

**Table 3:** Comparison of different fusion methods for orthopanchromatic RS image.

RS image fusion methods	Band	SD	MG	IE
The image fused with GPSA	R	59.2436	19.3854	7.4679
	G	59.6438	19.2543	7.6235
	B	59.7462	19.6273	7.6547
The image fused with DAA	R	58.7846	18.7659	8.7851
	G	58.6852	18.6953	8.6837
	B	58.5968	18.6538	8.6259
The image fused with YUV	R	59.4857	18.2467	8.5438
	G	59.5736	18.3574	8.6274
	B	59.6425	18.4369	8.7256
The image fused with IHS	R	59.7532	19.3258	8.6435
	G	60.3725	19.4321	8.7452
	B	60.4532	19.5643	8.8325
The image fused with DWT	R	61.5263	20.5836	7.6235
	G	61.6458	20.6235	7.7269
	B	61.7236	20.7354	7.8257
The image fused with AGA	R	62.6138	21.6438	9.6367
	G	62.7169	21.7261	9.7251
	B	62.8167	21.8145	9.8169
